# Functional Interplay between Type I and II Interferons Is Essential to Limit Influenza A Virus-Induced Tissue Inflammation

**DOI:** 10.1371/journal.ppat.1005378

**Published:** 2016-01-05

**Authors:** Sebastian A. Stifter, Nayan Bhattacharyya, Roman Pillay, Manuela Flórido, James A. Triccas, Warwick J. Britton, Carl G. Feng

**Affiliations:** 1 Immunology and Host Defense Group, Discipline of Infectious Diseases and Immunology, Sydney Medical School, The University of Sydney, Sydney, New South Wales, Australia; 2 Mycobacterial Research Program, The Centenary Institute, Camperdown, New South Wales, Australia; 3 Microbial Pathogenesis and Immunity Group, Discipline of Infectious Diseases and Immunology, Sydney Medical School, The University of Sydney, Sydney, New South Wales, Australia; 4 Discipline of Infectious Diseases and Immunology, Sydney Medical School, The University of Sydney, Sydney, New South Wales, Australia; 5 Department of Medicine, Sydney Medical School, The University of Sydney, Sydney, New South Wales, Australia; St. Jude Children's Research Hospital, UNITED STATES

## Abstract

Host control of influenza A virus (IAV) is associated with exuberant pulmonary inflammation characterized by the influx of myeloid cells and production of proinflammatory cytokines including interferons (IFNs). It is unclear, however, how the immune system clears the virus without causing lethal immunopathology. Here, we demonstrate that in addition to its known anti-viral activity, STAT1 signaling coordinates host inflammation during IAV infection in mice. This regulatory mechanism is dependent on both type I IFN and IFN-γ receptor signaling and, importantly, requires the functional interplay between the two pathways. The protective function of type I IFNs is associated with not only the recruitment of classical inflammatory Ly6C^hi^ monocytes into IAV-infected lungs, but also the prevention of excessive monocyte activation by IFN-γ. Unexpectedly, type I IFNs preferentially regulate IFN-γ signaling in Ly6C^lo^ rather than inflammatory Ly6C^hi^ mononuclear cell populations. In the absence of type I IFN signaling, Ly6C^lo^ monocytes/macrophages, become phenotypically and functionally more proinflammatory than Ly6C^hi^ cells, revealing an unanticipated function of the Ly6C^lo^ mononuclear cell subset in tissue inflammation. In addition, we show that type I IFNs employ distinct mechanisms to regulate monocyte and neutrophil trafficking. Type I IFN signaling is necessary, but not sufficient, for preventing neutrophil recruitment into the lungs of IAV-infected mice. Instead, the cooperation of type I IFNs and lymphocyte-produced IFN-γ is required to regulate the tissue neutrophilic response to IAV. Our study demonstrates that IFN interplay links innate and adaptive anti-viral immunity to orchestrate tissue inflammation and reveals an additional level of complexity for IFN-dependent regulatory mechanisms that function to prevent excessive immunopathology while preserving anti-microbial functions.

## Author Summary

Influenza A virus (IAV) is a leading cause of respiratory infection and induces a strong acute inflammation manifested by the recruitment of monocytes and neutrophils as well as the production of proinflammatory cytokines in infected lungs. The interferons (IFNs) are strongly induced by IAV and are known to mediate host resistance to the infection. However, in contrast to their well-studied inhibitory effect on viral replication, the effects of IFNs on host inflammatory responses are less well understood. In this manuscript, we demonstrate that anti-viral IFN signaling is also required for the orchestration of a tissue response associated with the protection against IAV infection in mice. Importantly, we identify that type I IFNs cross-regulate and cooperate with IFN-γ to inhibit monocyte activation and neutrophil infiltration, respectively. This study also demonstrates that Ly6C^lo^ monocytes/macrophages can potentially mediate influenza virus-induced inflammation, suggesting that IFNs dictate the homeostasis versus inflammatory function of mononuclear phagocytes in viral infection. Our study reveals a novel IFN-dependent regulatory mechanism designed to prevent the excessive immunopathology while preserving its anti-microbial functions. Moreover, these observations have particular relevance for understanding the mechanisms underlying the strong inflammatory response associated with lethal IAV strains and have implications for the development of new immunotherapies to treat influenza.

## Introduction

Influenza A virus (IAV) is a leading cause of respiratory infection and an ongoing threat to global health. Host clearance of IAV, which infects primarily airway epithelial cells, requires the development of both innate and adaptive immune responses [1,2]. Interestingly, recent studies have suggested that the host immune response rather than the cytopathic effect of viral infection plays the key role in driving tissue pathology and host mortality [3–5].

IAV triggers an acute pulmonary inflammation associated with the recruitment of inflammatory monocytes and neutrophils in infected lungs (reviewed in [6]). While it is clear that elevated neutrophil accumulation into infected lungs is associated with increased mortality following IAV infection [7,8], monocyte recruitment can be host protective or detrimental [9,10], suggesting that monocytes may play a multifactorial role in the infection. The current understanding of monocytes suggests that there are at least two major subsets: classical Ly6C^hi^ and nonclassical Ly6C^lo^ monocytes [11]. The classical Ly6C^hi^ monocytes are known to mediate various inflammatory conditions [12] and accumulate in large numbers in IAV-infected lungs [13]. In contrast, nonclassical Ly6C^lo^ cells have been shown to “patrol” the vasculature to clear damaged endothelial cells and contribute to tissue remodeling during the resolution phase of inflammation [14]. Interestingly, the patrolling Ly6C^lo^ monocytes have been shown to differentiate into alternatively activated macrophages during *Listeria monocytogenes* infection [14]. However, the phenotype and function of Ly6C^lo^ monocytes / macrophages (Mo/Mϕ) in IAV infection are currently unknown.

In addition to recruiting myeloid cells, IAV induces the production of proinflammatory cytokines, including type I and II interferons (IFNs), in infected animals. The type I IFN family consists of ~20 different members believed to be important in anti-viral and cancer immunity [15], whereas the sole member of type II IFN, IFN-γ, plays a major role in activating Mo/Mϕ and protection against intracellular bacterial and parasitic infections (Reviewed in [16]). In contrast to its extensively studied function in initiating a cell-autonomous anti-viral state, the mechanisms by which IFN signaling regulates host tissue responses to IAV infection are poorly understood. Several recent studies have shown that viral-induced type I IFNs promote the accumulation of classical Ly6C^hi^ monocytes into the airway and lungs of IAV-infected mice [17,18]. However, it is unclear whether these cytokines also regulate the function of pulmonary monocytes for the resistance to IAV infection. Moreover, the contribution of IFN-γ to IAV-induced pulmonary tissue inflammation is not clearly defined.

We report in this study that type I IFN and IFN-γ signaling each play a pleiotropic role in the pulmonary inflammatory response to IAV. Importantly, IFN cross-regulation and cooperation are essential for the suppression of monocyte- and neutrophil-driven tissue inflammation. While antagonizing IFN-γ signaling to inhibit Mo/Mϕ activation, type I IFNs synergize with IFN-γ to inhibit neutrophil infiltration. Moreover, we demonstrate that in the absence of type I IFN signaling, Ly6C^lo^ Mo/Mϕ become more proinflammatory than their Ly6C^hi^ counterparts. Since the Ly6C^lo^ Mo/Mϕ population is traditionally associated with tissue remodelling rather than inflammation, our findings also reveal an unrecognized pro-inflammatory potential for Ly6C^lo^ Mo/Mϕ and suggest that IFNs dictate the homeostasis versus inflammatory function of mononuclear cells in viral infection.

## Results

### STAT1 signaling controls pulmonary inflammatory response to IAV infection

**Fig 1:**
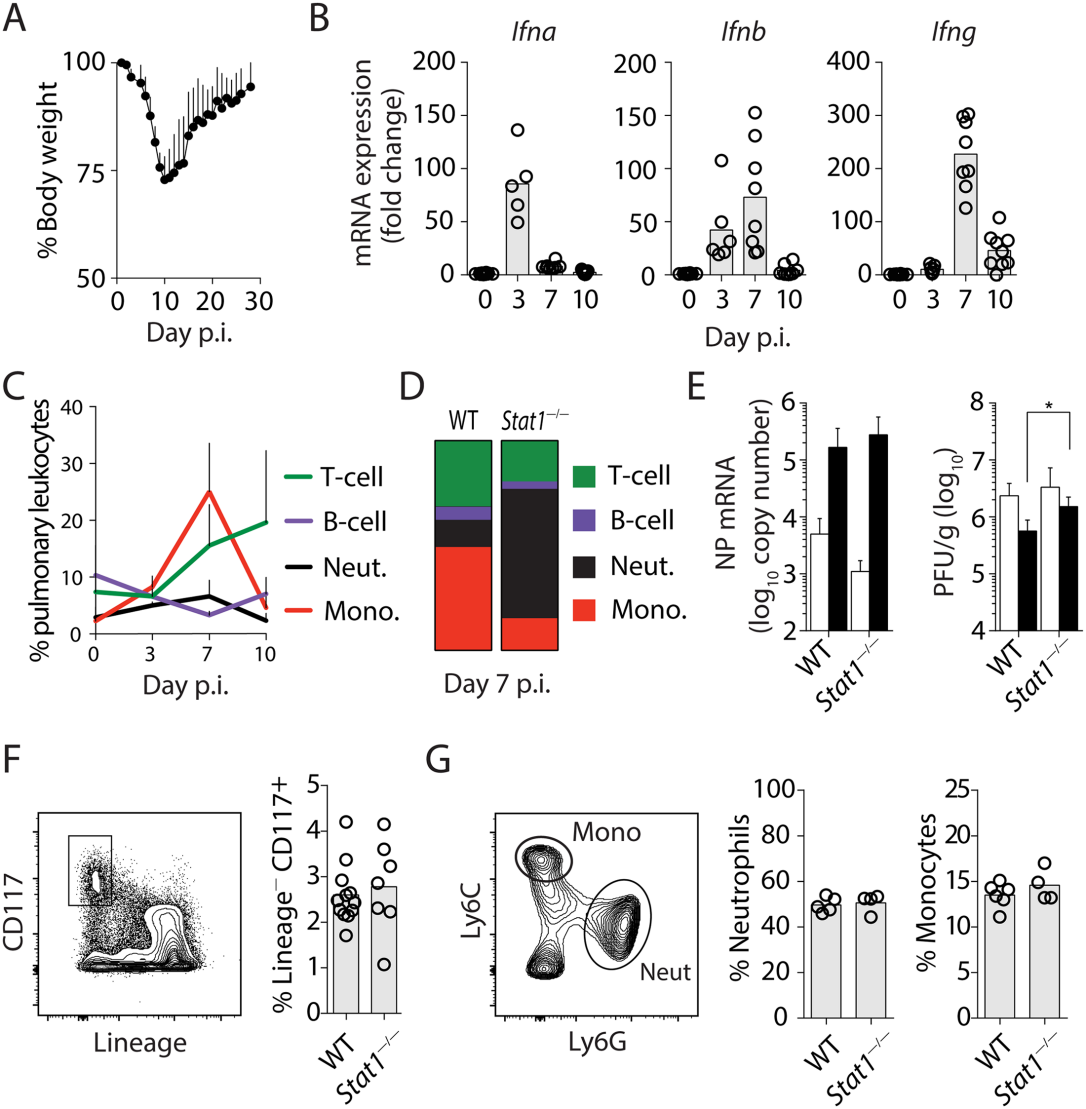
STAT1 signaling dictates host cellular inflammatory response to IAV independent of regulation of myelopoiesis. **(A)** Change in body weight following infection with IAV. Mice were infected i.n. with IAV PR8 (20 PFU) and body weight monitored daily. Data shown are mean weight ± SD and are representative of 2 independent experiments (5 mice / experiment). **(B)** mRNA expression of *Ifna*, *Ifnb* and *Ifng* in lungs of infected mice was analyzed using qRT-PCR at the indicated time points. Data shown are mean fold increase relative to uninfected controls. Data were pooled from 2 independent experiments with similar trend (n = 5–9 mice per time point). Circles denote individual mice and bars represent group means. **(C)** Proportion of lung leukocytes following IAV infection. Data shown are mean ± SD (n = 4) and are representative of at least 3 independent experiments. **(D)** Vertical slice bars indicate the relative proportions of the major lung leukocyte subsets quantified using flow cytometric analysis in infected WT and *Stat1* ^—/—^mice at d7 p.i.. The data shown are the mean of three mice and are representative of 3 independent experiments. **(E)** Total IAV NP mRNA copy number and PFU in lungs of infected mice at d3 (open bars) and d7 (closed bars) p.i. as determined by qRT-PCR and MDCK plaque assay, respectively. Data shown are mean copy numbers ± SD or mean PFU ± SD and were pooled from 3 independent experiments (n = 3–11 mice per group). **(F and G)** Representative flow cytometry plots and summary data depicting the proportions of **(F)** hematopoietic stem / progenitors (Lineage^—^CD117^+^) as well as **(G)** mature monocytes (Mono, CD11b^+^Ly6C^+^Ly6G^—^) and neutrophils (Neut, CD11b^+^Ly6G^+^Ly6C^—^) in the BM of infected WT and *Stat1* ^—/—^mice at d7 p.i.. Circles denote individual mice and bars represent group means. Data were pooled from 2 independent experiments (n = 4–6 mice).

Sub-lethal, intranasal (i.n.) infection with influenza A/Puerto Rico/8 (PR8) virus in wild-type (WT) mice is characterized by a progressive weight loss that peaks at day 10, after which mice recover and clear the infection (Fig 1A and [19]). To investigate the function of IFNs in shaping the host response to IAV infection, we first examined IFN gene expression and observed that while in WT mice type I IFNs *Ifna* and *Ifnb* were both rapidly up-regulated at day 3 post-infection (p.i.), the sole type II IFN, *Ifng*, was not significantly induced until day 7 p.i. (Fig 1B). The expression of both type I and II IFNs declined to baseline levels by day 10 p.i.. In WT mice, the cellular immune response to IAV infection was characterized by the strong influx of monocytes that peaked at day 7 and then declined by day 10, at which point T cells became the dominant immune cell subset in lungs (Fig 1C). In contrast to WT controls, *Stat1*-deficient mice, lacking both type I IFN and IFN-γ signaling, displayed a neutrophil-enriched cellular response (Fig 1D), highlighting the critical importance of IFN signaling in orchestrating a protective tissue response to IAV infection. Next, we quantified the viral loads at days 3 and 7 p.i. using both qRT-PCR and classical plaque forming (PFU) assays. While mRNA copy numbers of viral nucleoprotein (NP) determined by qRT-PCR were comparable in WT and *Stat1* ^—/—^mice, the latter animals showed a marginal increase (0.4 log) in PFUs at day 7 (Fig 1E). The extent of defect in viral control in *Stat1* ^—/—^mice observed is consistent with that reported previously [20,21], suggesting that the dysregulated tissue response in *Stat1* ^—/—^mice cannot be explained fully by increased viral loads.

To determine whether the altered pulmonary inflammation in infected *Stat1* ^—/—^mice stems from dysregulated myelopoiesis in bone marrow (BM) or cell trafficking in the periphery, or both, we analyzed hematopoietic cells in the BM of infected WT and *Stat1* ^—/—^mice by flow cytometry. The stem/progenitor cell populations are commonly defined as lineage-negative Sca1^+^cKit^+^ (CD117^+^) (LSK) cells [22,23]. However, since Sca1 expression is regulated by IFNs (S1 Fig and [22]), we omitted Sca1 staining in the analysis. We did not observe a significant difference in the percentage of lineage-negative CD117^+^ progenitor cells (Fig 1F), or mature BM-residing CD11b^+^Ly6G^+^Ly6C^lo^ neutrophils and CD11b^+^Ly6G^—^Ly6C^hi^ monocytes (Fig 1G) in infected WT and *Stat1* ^—/—^mice, suggesting that IFN signaling plays a minimal role in regulating central myelopoiesis in the BM during IAV infection.

### Cell-intrinsic type I IFN signaling promotes the recruitment of inflammatory Ly6C^hi^ monocytes into IAV-infected lungs

**Fig 2:**
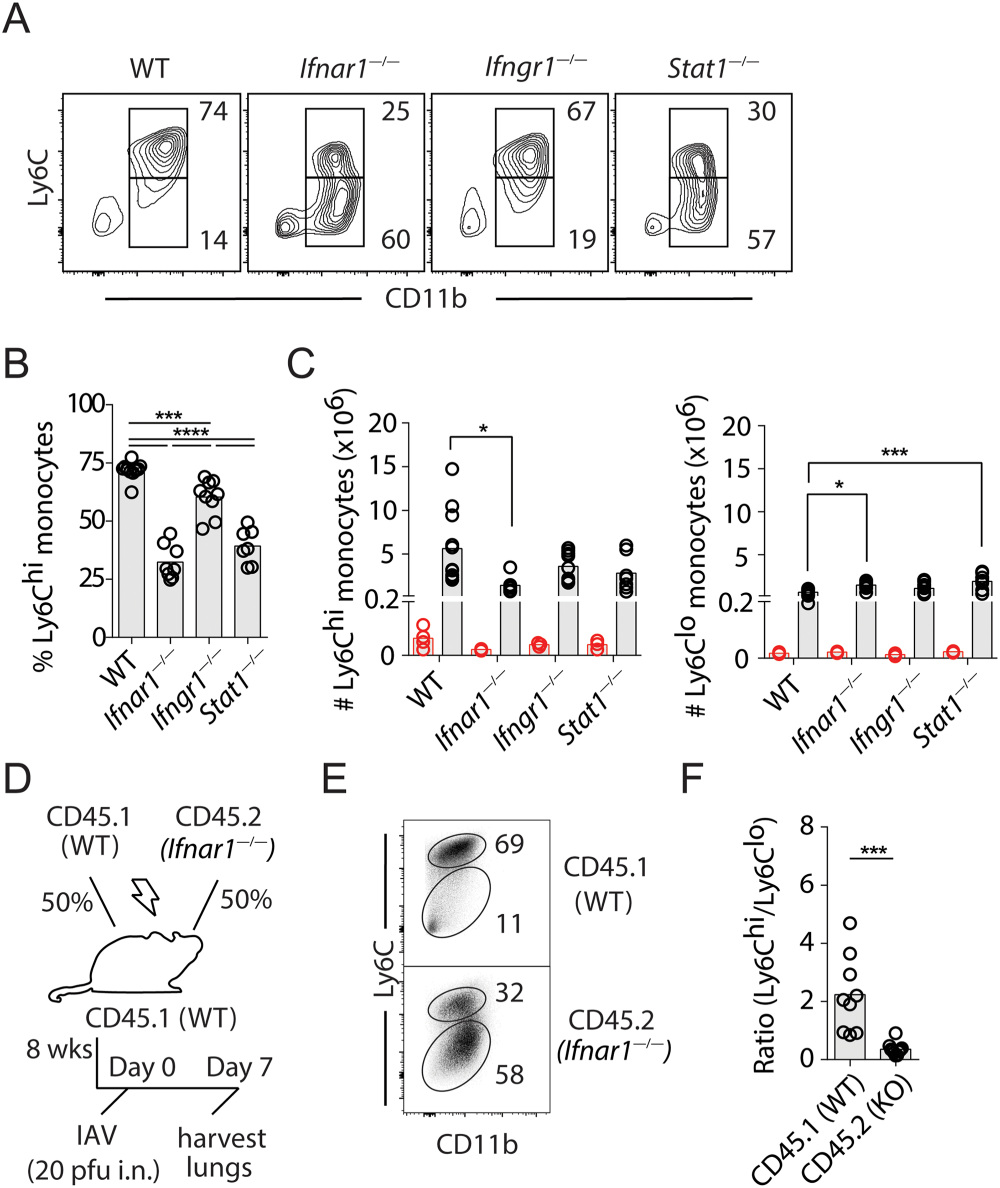
Type I IFNs signal directly in monocytes to promote their migration to IAV-infected lungs. Mice were infected with IAV and lung single cell suspensions analyzed d7 p.i. by flow cytometry. The flow cytometry data shown are gated on CD4^—^CD8^—^B220^—^NK1.1^—^Ly6G^—^SiglecF^—^cell populations. **(A)** Representative flow cytometry plots depicting the percentage of Ly6C^hi^ and Ly6C^lo^ Mo/Mϕ in the lungs of infected WT, *Ifnar1* ^—/—^, *Ifngr1* ^—/—^or *Stat1* ^—/—^mice. **(B)** Summary data showing the percentage of Ly6C^hi^ monocytes in infected lungs. Data were pooled from at least 3 independent experiments (n = 6–10 mice / group). **(C)** Summary data showing the total number of Ly6C^hi^ and Ly6C^lo^ cell populations in infected lungs at d3 (red circles) and d7 (black circles) p.i.. Data were pooled from at least 3 independent experiments (n = 6–10 mice / group). **(D)** Schematic illustration of the strategy used for the generation, infection and analysis of mixed BM chimeric mice. **(E)** Representative flow cytometry plots demonstrating the percentage of pulmonary Ly6C^hi^ and Ly6C^lo^ Mo/Mϕ in CD45.1^+^ (WT) and CD45.2^+^ (*Ifnar1* ^—/—^) compartments of infected mixed BM chimera mice. **(F)** Summary analysis of the ratio of Ly6C^hi^ to Ly6C^lo^ subsets in CD45.1^+^ (WT) and CD45.2^+^ (*Ifnar1* ^—/—^) compartments. Data are representative of 4 independent experiments (n = 7–9 mice). In **B**, **C** and **F**, circles denote individual mice and bars represent group means.

To determine the relative contribution of type I and II IFNs to monocyte recruitment in IAV infection, we analyzed monocyte populations in the lungs of infected WT, *Ifnar1* ^*—/—*^, *Ifngr1* ^*—/—*^ *and Stat1* ^*—/—*^mice using flow cytometry. When compared to WT mice, *Ifnar1* ^—/—^ and *Stat1* ^*—/—*^mice displayed a significant reduction in CD11b^+^Ly6C^hi^ monocytes in the lungs at day 7 p.i. (Fig 2A, 2B and 2C). The difference is unlikely due to kinetic variations of the pulmonary response to IAV in IFN signaling-sufficient and deficient mice, as the significant influx of leukocytes into the infected lungs was not observed until day 7 p.i. in all groups (Fig 2B and 2C). In contrast to *Ifnar1* ^*—/—*^animals, monocyte recruitment in *Ifngr1* ^*—/—*^mice was largely unaffected, suggesting that type I IFN signaling alone is sufficient to trigger monocyte recruitment into infected lungs. We next determined whether type I IFNs act directly or indirectly on monocyte populations to regulate their trafficking to lungs. Lethally irradiated CD45.1^+^ WT recipient mice were reconstituted with equal numbers of CD45.1^+^ WT and CD45.2^+^ *Ifnar1* ^*—/—*^BM cells. After full reconstitution (8–10 wk), chimera mice were infected with IAV and the cellular response analyzed at day 7 p.i. (Fig 2D). We found that the defect in the recruitment of Ly6C^hi^ inflammatory monocytes observed in *Ifnar1* ^*—/—*^mice was not restored in CD45.2^+^ *Ifnar1* ^*—/—*^cells in mixed *Ifnar1* ^*—/—*^and *Ifnar1* ^*+/+*^ BM chimeric mice (Fig 2E). As such, the ratio of Ly6C^hi^ / Ly6C^lo^ monocytes in the CD45.2^+^ *Ifnar1* ^*—/—*^compartment was significantly lower than that in the CD45.1^+^ *Ifnar1* ^*+/+*^ compartment, indicating that direct type I IFN signaling in Ly6C^hi^ monocytes is required for their recruitment into infected lungs (Fig 2F).

### Type I IFNs regulate the phenotype and function of pulmonary monocytes in IAV infection

**Fig 3:**
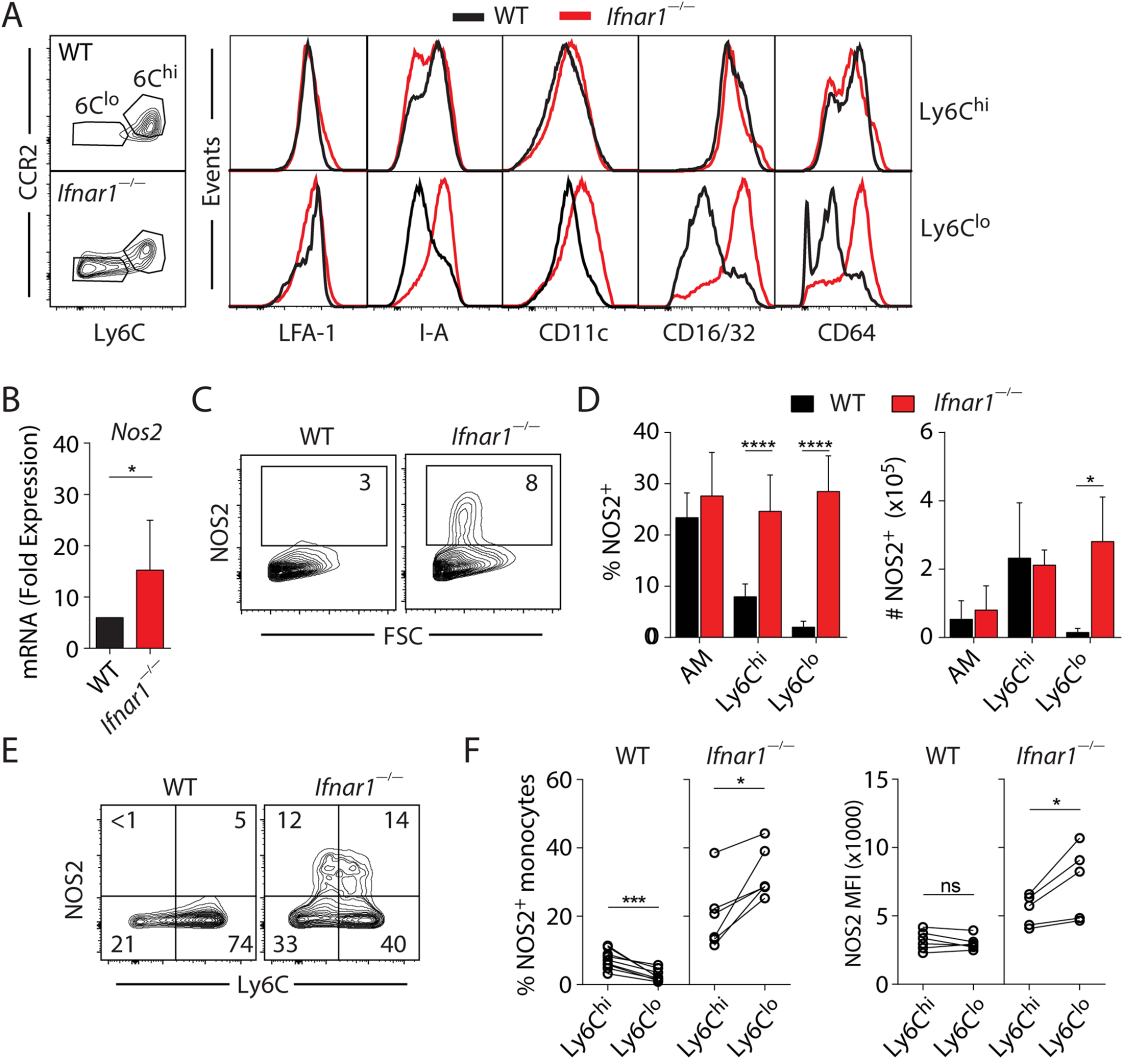
Type I IFNs regulate the phenotype and effector functions of Mo/Mϕ in IAV-infected lungs. **(A)** Representative flow plots demonstrating Ly6C^hi^ or Ly6C^lo^ mononuclear phagocyte populations and histograms depicting the expression of surface molecules on Ly6C^hi^ or Ly6C^lo^ cell subsets in the lungs of d7 infected WT and *Ifnar1* ^—/—^mice (n = 7 mice / genotype). The flow cytometry data shown are gated on CD11b^+^CD4^—^CD8^—^B220^—^NK1.1^—^Ly6G^—^SiglecF^—^cell populations. **(B)** *Nos2* expression in lungs of infected mice determined at d7 p.i. using qRT-PCR. Data shown are mean fold increases (relative to uninfected controls) ± SD. Data were pooled from 3 independent experiments (n = 6–9 mice / genotype). **(C)** Representative flow plots of intracellular NOS2 expression in the lungs of infected WT and *Ifnar1* ^—/—^mice at d7 p.i.. The flow cytometry data shown are gated on live, singlet cells. Flow plots are representative of 3 independent experiments with n = 6–9 mice / genotype. **(D)** Percentage and number of NOS2-positive mononuclear cells in the lungs of infected WT and *Ifnar1* ^—/—^mice quantified using flow cytometry. Data were pooled from 3 independent experiments with a similar trend and are mean levels ± SD (n = 6–9 mice / genotype). **(E)** Representative flow plots depicting NOS2 present in Ly6C^hi^ and Ly6C^lo^ cells. The flow cytometry data shown are gated on CD11b^+^CD4^—^CD8^—^B220^—^NK1.1^—^Ly6G^—^SiglecF^—^cell populations. **(F)** Paired analysis of percentage and mean fluorescence intensity (MFI) of NOS2 in Ly6C^hi^ and Ly6C^lo^ populations in WT and *Ifnar1* ^—/—^mice at d7 p.i.. Data were pooled from 3 independent experiments and each paired data set represents an individual mouse. Statistical analyses were performed using paired Student’s *t*-test.

We next examined whether type I IFN signaling regulates the phenotype and function of major populations of CD11b^+^ monocytes in IAV-infected lungs. Consistent with current knowledge of mononuclear phagocyte subsets [11], we observed that Ly6C^hi^ monocytes in IAV-infected lungs displayed higher CCR2 expression than their Ly6C^lo^ counterparts in WT and *Ifnar1* ^—/—^mice (Fig 3A). Furthermore, expression of the integrin LFA-1 (CD11a), a key molecule responsible for the function of monocytes [24], was unchanged on either Ly6C^hi^ or Ly6C^lo^ cells irrespective of type I IFN signaling. Strikingly, however, loss of type I IFN signaling on Ly6C^lo^ but not Ly6C^hi^ populations resulted in significant increases in the expression of MHC-II (I-A), CD11c, CD16/32 and CD64, suggesting that in *Ifnar1* ^*—/—*^mice, Ly6C^lo^ Mo/Mϕ display a phenotype that is characteristic of activated proinflammatory monocytes. Consistent with this hypothesis, we found that *Nos2*, a known proinflammatory molecule produced predominantly by monocytes, was more highly expressed in the lungs of *Ifnar1* ^*—/—*^mice than WT animals (Fig 3B), despite the reduction in Ly6C^hi^ monocytes in *Ifnar1* ^—/—^mice (Fig 2B).

Consistent with *Nos2* gene analysis, flow cytometric analysis revealed that there was a significant increase in NOS2-expressing cells in the lungs of infected *Ifnar1* ^*—/—*^mice compared to WT animals (Fig 3C). Further analysis revealed that multiple mononuclear cells in IAV-infected lungs produced NOS2 (Fig 3D). However, while Ly6C^hi^ monocytes were the major NOS2-producing cells in WT animals, both Ly6C^hi^ and Ly6C^lo^ subsets contributed to the NOS2 production in the lungs of infected *Ifnar1* ^*—/—*^mice (Fig 3D and 3E). Paired analysis of the two monocyte subsets among individual mice demonstrated that in *Ifnar1* ^*—/—*^animals, both the percentage of cells expressing NOS2 as well as the total quantity of NOS2 produced per cell was higher in Ly6C^lo^ than Ly6C^hi^ monocytes, suggesting that the former subset is more susceptible to type I IFN-dependent suppression (Fig 3F). Therefore, type I IFNs are able to regulate not only trafficking, but also the phenotype and effector function of mononuclear cells in the lung during IAV infection.

### Type I IFNs suppress IFN-γ-dependent monocyte activation by down-regulating IFN-γ receptor

**Fig 4:**
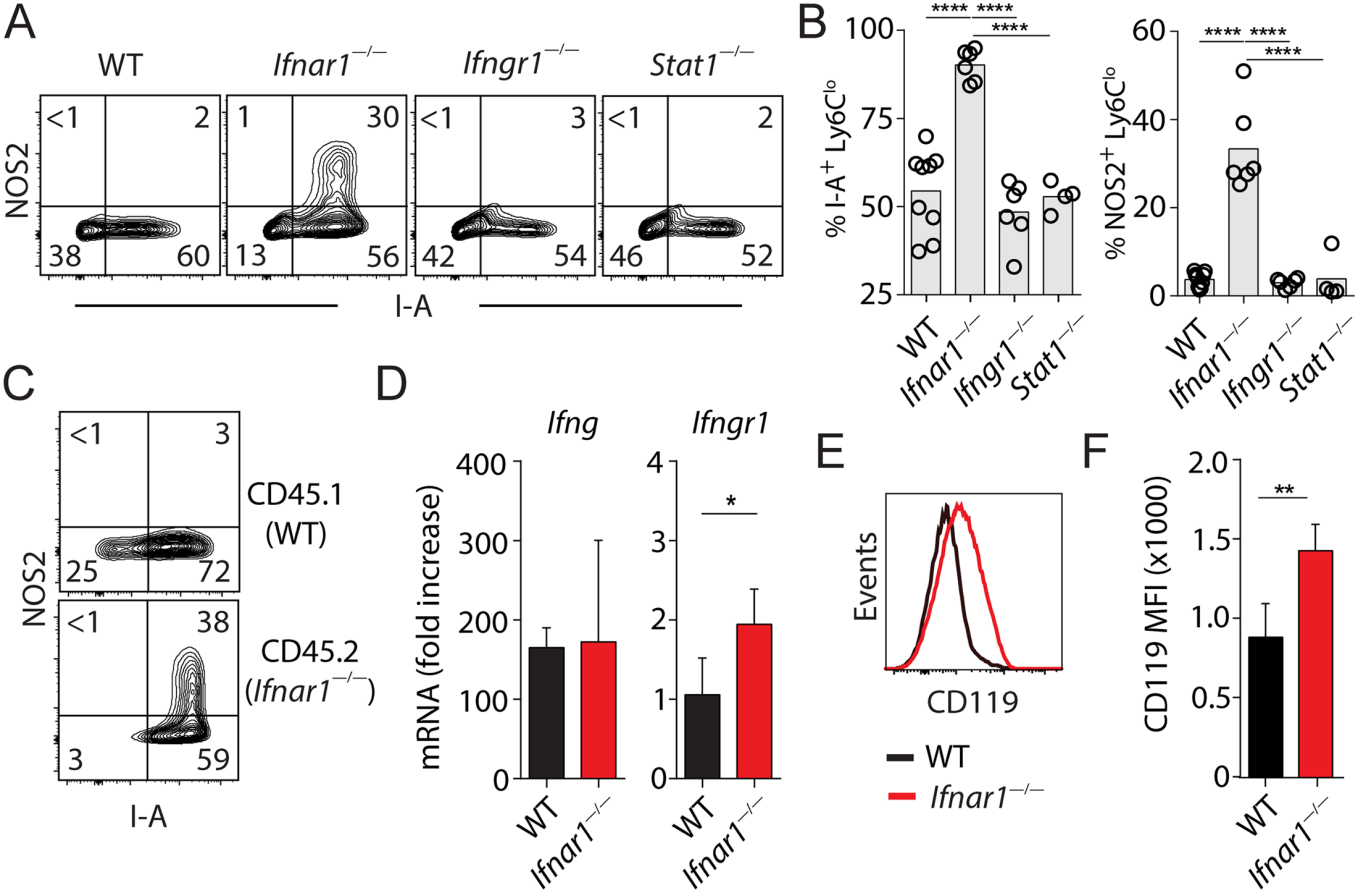
Type I IFNs protect Ly6C^lo^ Mo/Mϕ from IFN-γ-induced activation by down-regulating IFN-γ receptor. **(A)** Representative flow cytometry plots and **(B)** summary statistics of I-A and NOS2 expression in Ly6C^lo^ Mo/Mϕ. The flow cytometry data shown are gated on CD11b^+^CD4^—^CD8^—^B220^—^NK1.1^—^Ly6G^—^SiglecF^—^cell populations. Circles in **(B)** denote individual mice and bars represent group means. Data were pooled from 3 independent experiments with a total of 4–9 mice per group. **(C)** Representative flow plots showing the percentage of NOS2 and I-A positive CD45.1^+^ (WT) and CD45.2^+^ (*Ifnar1* ^—/—^) Ly6C^lo^ Mo/Mϕ of infected mixed BM chimera mice at d7 p.i.. The flow cytometry data shown are gated on CD11b^+^CD4^—^CD8^—^B220^—^NK1.1^—^Ly6G^—^SiglecF^—^cell populations. The mixed BM chimeric mice were generated, infected and analyzed as described in Fig 2D. Data are representative of 2 independent experiments (n = 5). **(D)** *Ifng* and *Ifngr1* mRNA expression in lungs of infected mice at d7 p.i. were analyzed using qRT-PCR. Data shown are mean fold increases (relative to uninfected controls) ± SD. Data were pooled from 3 independent experiments (n = 6–9 mice / genotype). **(E)** Representative flow cytometric histogram of IFN-γ receptor 1 (CD119) expression on Ly6C^lo^ Mo/Mϕ and **(F)** mean fluorescence intensity of CD119 expression (± SD) on Ly6C^lo^ Mo/Mϕ of infected WT and *Ifnar1* ^—/—^mice at d7 p.i.. Data were pooled from 2 independent experiments with a total of 5–6 mice per genotype.

Since the surface markers and NOS2 examined above are known to be highly sensitive to induction by IFN-γ [25–27], we suspected that IFN-γ might play a role in the up-regulation of these molecules in the infected *Ifnar1* ^*—/—*^mice. Indeed, at day 3 p.i., a time point at which minimal levels of IFN-γ are produced (Fig 1B), there were no differences in the expression of I-A, CD11c or CD64 on Ly6C^hi^ and Ly6C^lo^ cells (S2 Fig). To demonstrate directly that IFN-γ is responsible for the up-regulation of the molecules, we compared the expression of I-A and NOS2 in Ly6C^lo^ Mo/Mϕ of WT, *Ifnar1* ^*—/—*^, *Ifngr1* ^*—/—*^and *Stat1* ^*—/—*^mice at day 7 following IAV infection. We found that the enhanced expression of I-A and NOS2 observed in lungs of *Ifnar1* ^*—/—*^animals was completely abolished in *Ifngr1* ^—/—^mice as well as *Stat*1^—/—^mice that lack both type I IFN and IFN-γ signaling pathways (Fig 4A and 4B), suggesting that up-regulation of I-A and NOS2 in the absence of type I IFN signaling is driven by IFN-γ.

To determine whether type I IFNs act directly on monocytes to exert their suppressive effect, we analyzed NOS2 production in IAV-infected, mixed *Ifnar1* ^*+/+*^ and *Ifnar1* ^*—/—*^BM chimera mice and found that type I IFNs signal directly to suppress NOS2 production in both Ly6C^lo^ (Fig 4C) and Ly6C^hi^ (S3A Fig) mononuclear cells. Moreover, as we observed previously in *Ifnar1* ^*—/—*^mice (Fig 3F), Ly6C^lo^ Mo/Mϕ in BM chimera mice expressed higher levels of NOS2 than their Ly6C^hi^ counterparts (S3B Fig), confirming that enhanced susceptibility to type I IFN inhibition is intrinsic to this Mo/Mϕ subset. We next determined whether the enhanced IFN-γ-inducible response in IAV-infected *Ifnar1* ^*—/—*^mice was a consequence of increased IFN-γ production or signaling by examining the expression of *Ifng* and *Ifngr1* in infected lungs. We found that although *Ifng* expression was comparable in the lungs of infected WT and *Ifnar1* ^*—/—*^mice, *Ifngr1* levels were significantly higher in *Ifnar1* ^—/—^animals (Fig 4D), suggesting that enhanced IFN-γ signaling rather than IFN-γ production is responsible for the increased IFN-γ-inducible response in *Ifnar1* ^*—/—*^mice. To test this hypothesis, we analyzed the cell surface expression of IFN-γ receptor 1 (CD119) by flow cytometry and found CD119 expression was significantly increased on Ly6C^lo^ Mo/Mϕ of *Ifnar1* ^—/—^mice compared to WT mice (Fig 4E and 4F). Therefore, the intrinsic regulation of IFN-γ receptor levels by type I IFNs appears to be a mechanism by which Mo/Mϕ activation is suppressed in the presence of type I IFNs.

### Type I IFN signaling is necessary but not sufficient for the inhibition of neutrophil migration into the lungs of IAV-infected mice

**Fig 5:**
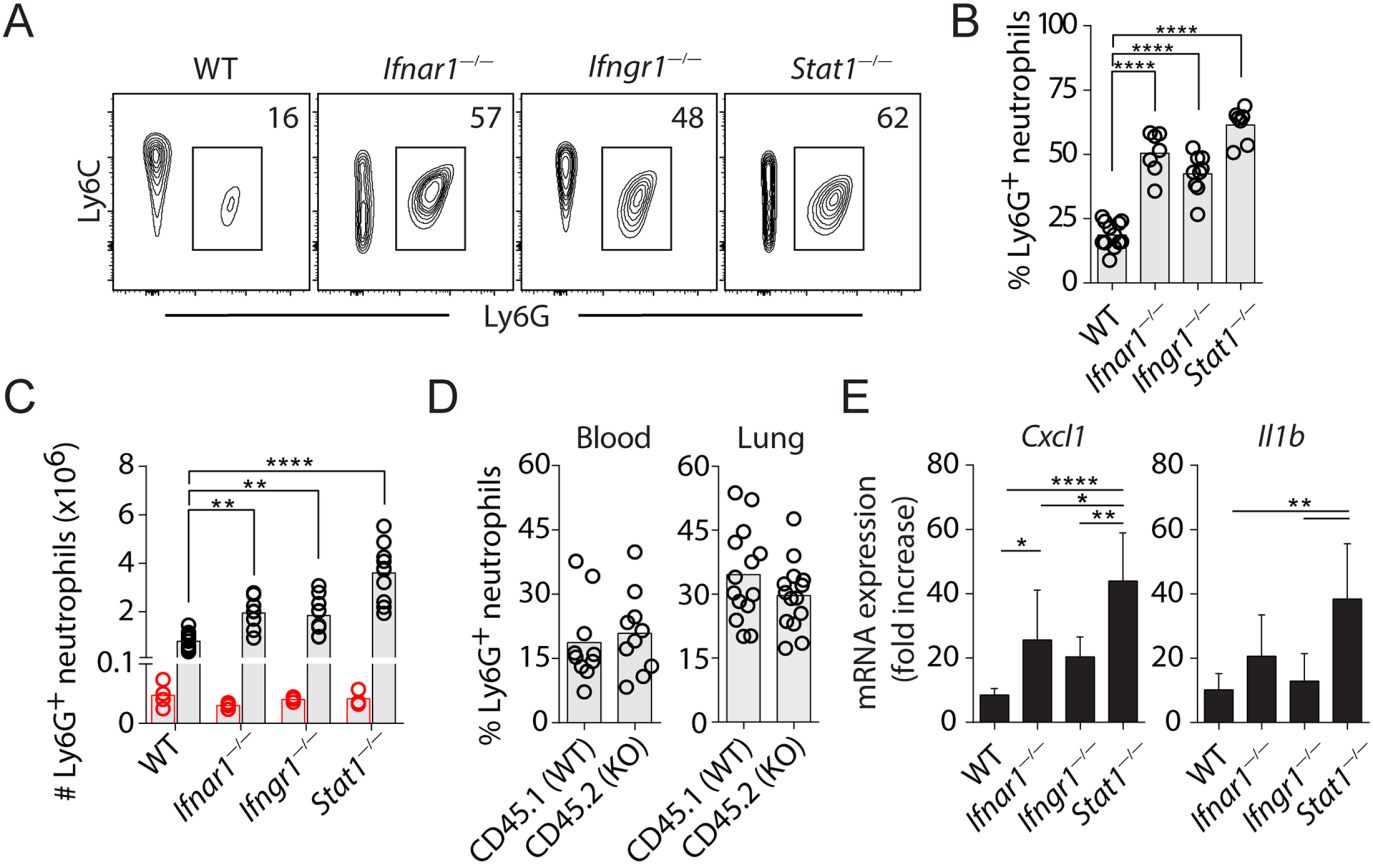
Type I IFN and IFN-γ signaling synergize to inhibit neutrophil trafficking to IAV-infected lungs. **(A)** Representative flow plots depicting lung Ly6G^+^ neutrophil populations. The flow cytometry data shown are gated on CD11b^+^CD4^—^CD8^—^B220^—^NK1.1^—^SiglecF^—^cell populations. **(B)** The percentage of neutrophils in WT, *Ifnar1* ^—/—^, *Ifngr1* ^—/—^or *Stat1* ^—/—^mice determined by flow cytometry at d7 p.i.. **(C)** The number of neutrophils in WT, *Ifnar1* ^—/—^, *Ifngr1* ^—/—^or *Stat1* ^—/—^mice determined by flow cytometry at d3 (red circles) and d7 (black circles) p.i.. Data were pooled from 2 independent experiments. **(D)** Percentage of Ly6G^+^ neutrophils in the blood and lungs of infected mixed BM chimera mice. The mixed CD45.1^+^ (WT) and CD45.2^+^ (*Ifnar1* ^—/—^) BM chimeric mice were generated, infected and analyzed using flow cytometry as described in Fig 2D. The data show the percentage of Ly6G^+^ neutrophils among CD45.1^+^ (WT) or CD45.2^+^ (*Ifnar1* ^—/—^) cells in the blood or lung of infected mixed BM chimeric mice at d7 p.i.. Data are representative of 2 independent experiments (n = 10–14 mice / study). **(E)** mRNA expression of *Cxcl1* and *Il1b* in lungs analyzed using qRT-PCR at d7 p.i.. Data shown are mean fold increases (relative to uninfected controls) ± SD. Data were pooled from 3 independent experiments with a similar trend. In **B**, **C** and **D**, circles denote individual mice and bars represent group means.

Our findings above suggest that type I IFN signaling is dominant over IFN-γ in the recruitment of Ly6C^hi^ monocytes to the lungs of IAV-infected mice (Fig 2). To investigate the relative contribution of type I and type II IFNs on the STAT1-dependent suppression of neutrophil migration (Fig 1D), WT, *Ifnar1* ^*—/—*^, *Ifngr1* ^*—/—*^and *Stat1* ^*—/—*^mice were infected with IAV and the relative abundance of neutrophils in each mouse strain determined by flow cytometry. Interestingly, we observed that both percentage and numbers of CD11b^+^Ly6G^+^ neutrophils were significantly elevated in the lungs of *Ifnar1* ^*—/—*^as well as *Ifngr1* ^*—/—*^mice compared to WT animals at day 7, but not day 3 p.i. (Fig 5A, 5B and 5C). Importantly, this defect was further exaggerated in *Stat1* ^*—/—*^mice, suggesting a synergistic function of type I and II IFNs in suppressing neutrophil recruitment during influenza infection. Moreover, in contrast to the cell-intrinsic function of type I IFNs in monocyte migration described above (Fig 2D), we found that the percentage of CD11b^+^Ly6C^lo^Ly6G^+^ neutrophils was comparable among CD45.1^+^ WT and CD45.2^+^ *Ifnar1* ^*—/—*^compartments before (S4 Fig) and following (Fig 5D) infection, suggesting that type I IFNs regulate neutrophil migration through a cell-extrinsic manner. Therefore, type I IFNs employ distinct mechanisms to regulate monocyte and neutrophil trafficking in IAV infection and act in concert with IFN-γ to prevent accumulation of tissue-damaging neutrophils at the site of infection. To investigate potential cell-extrinsic mechanisms responsible for the increased accumulation of neutrophils, we measured gene expression of the known neutrophil-attracting chemokine *Cxcl1* and the cytokine *Il1b* in WT and IFN-signaling deficient animals. *Stat1* ^*—/—*^mice displayed significantly higher expression of *Cxcl1* and *Il1b* than in either *Ifnar1* ^*—/—*^or *Ifngr1* ^*—/—*^mice (Fig 5E), consistent with the notion that IFNs synergize to suppress neutrophil chemotactic chemokine/cytokine production in IAV infection.

### Adaptive IFN-γ and type I IFNs cooperate to inhibit neutrophil trafficking into lungs of IAV-infected mice

**Fig 6:**
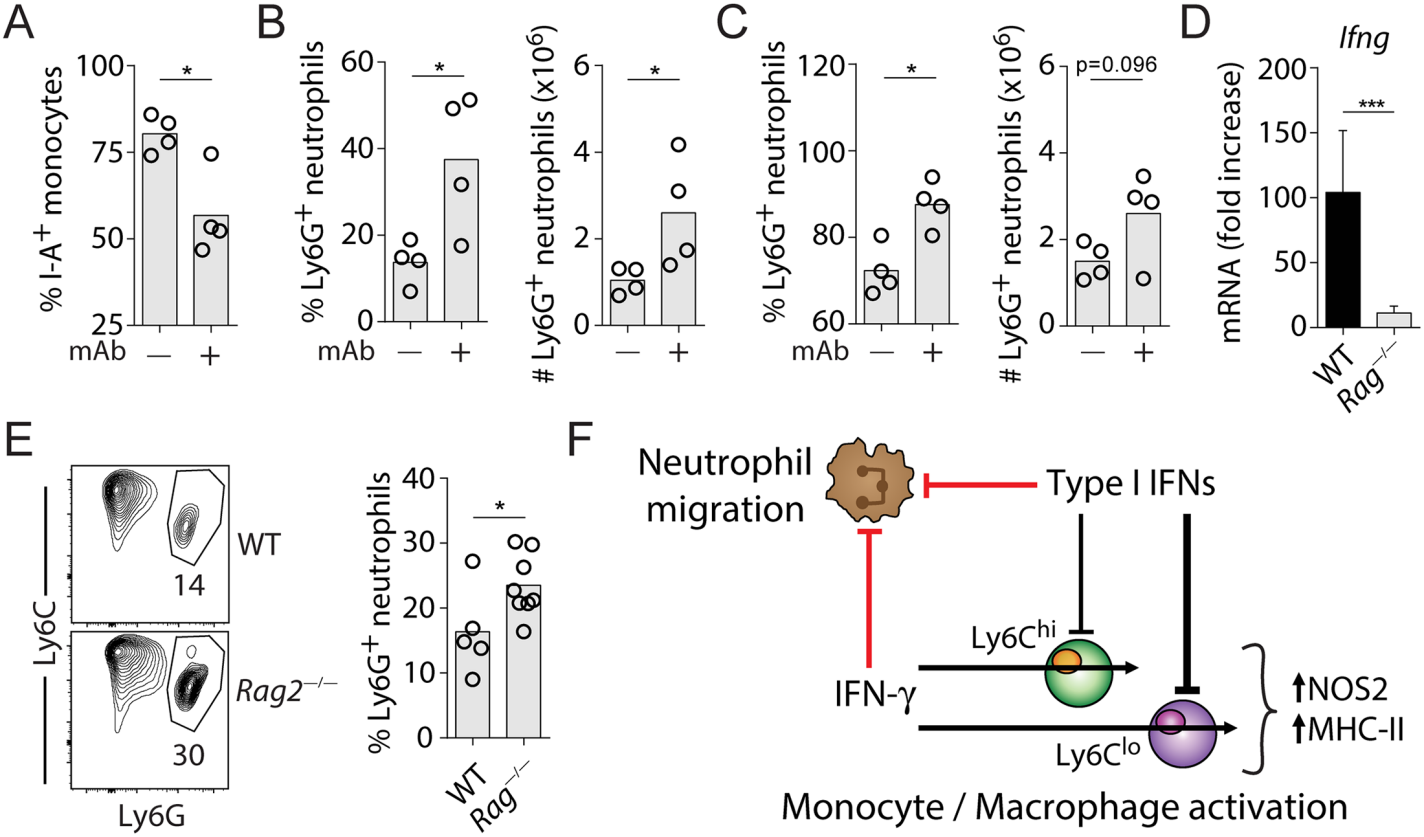
IFN-γ signaling inhibits neutrophil trafficking to IAV-infected lungs independent of type I IFNs and inflammatory monocytes. **(A–C)** Lethally irradiated CD45.1^+^ WT mice were reconstituted with BM cells from CD45.2^+^ *Ifnar1* ^—/—^mice and infected with IAV. Mice were injected i.p. with 500 μg anti-IFN-γ neutralizing mAb on d2 and d6 of IAV infection or left untreated. Lung tissues and single cell suspensions were analyzed at d7 p.i. using flow cytometry. **(A)** Percentage of I-A^+^ Mo/Mϕ, **(B and C)** Percentage and total number of Ly6G^+^ neutrophils in **(B)** lungs and **(C)** BAL of untreated or anti-IFN-γ mAb treated chimera mice. Data are representative of 2 independent experiments. Circles denote individual mice and bars represent group means. **(D–F)** WT and *Rag2* ^—/—^mice were infected with IAV and analyzed d7 p.i.. **(D)** mRNA expression of *Ifng* in lungs was analyzed using qRT-PCR at d7 p.i.. Data shown are mean fold increases (relative to uninfected controls) ± SD. Data were pooled from 3 independent experiments (n = 6–7 mice / genotype). **(E)** Representative flow cytometry plots and summary data depicting the percentage of Ly6G^+^ neutrophils in the lungs of IAV-infected WT and *Rag2* ^—/—^mice d7 p.i. Data were pooled from 2 independent experiments with a total of 5–8 mice per genotype. Circles denote individual mice and bars represent group means. The flow cytometry data shown are gated on CD11b^+^CD4^—^CD8^—^B220^—^NK1.1^—^SiglecF^—^cell populations. **(F)** Schematic diagram illustrating the functional intersecting points between type I IFN and IFN-γ signaling pathways in IAV infection. Type I IFNs co-operate with IFN-γ to suppress neutrophil accumulation at the site of infection (red lines). Type I IFNs antagonize IFN-γ signaling to inhibit Mo/Mϕ activation (black lines).

STAT1 is one of many transcription factors that can transduce both type I IFN and IFN-γ receptor signaling, but it can also mediate IFN-independent functions [28–30]. Therefore, it is possible that the increased neutrophilic influx in IAV-infected *Stat1* ^*—/—*^mice, as compared to *Ifnar1* ^*—/—*^mice, is independent of IFN-γ signaling. Moreover, it is unclear whether IFN-γ-dependent neutrophil suppression requires intact type I IFN signaling. Type I IFNs are known to regulate neutrophil recruitment by signaling directly in inflammatory monocytes to suppress their production of neutrophil chemoattracting chemokine *Cxcl2* [31]. To address these questions, we generated *Ifnar1* ^*—/—*^BM reconstituted WT chimera mice, which are deficient in type I IFN signaling in hematopoietic cells only. Similar to *Ifnar1* ^*—/—*^mice, these chimeric mice also displayed defective pulmonary accumulation of Ly6C^hi^ monocytes following IAV infection (S5 Fig). *Ifnar1* ^*—/—*^BM chimera mice treated with anti-IFN-γ antibody at day 2 and 6 p.i. exhibited a marked reduction in I-A expression on monocytes when analyzed at day 7 (Fig 6A). This is consistent with our previous observation that IFN-γ augments MHC class II expression (Fig 4) and confirms that the mAb administration successfully blocked IFN-γ activity. Importantly, consistent with the results presented in Fig 5, we found both the proportion and numbers of neutrophils to be significantly elevated in the anti-IFN-γ antibody-treated mice compared to untreated animals (Fig 6B). We also enumerated neutrophils in the bronchoalveolar lavage (BAL) and observed a similar increase in neutrophils in the airway of anti-IFN-γ antibody treated mice when compared to untreated controls (Fig 6C), indicating that IFN-γ regulates neutrophil migration into both the lung parenchyma and bronchoaveolar air spaces independent of type I IFN signaling in inflammatory monocytes. These results suggest that IFN-γ produced by the adaptive immune response to IAV infection regulates the tissue inflammatory response in addition to its anti-viral activity [1]. To test this hypothesis, we infected WT and *Rag2* ^*—/—*^mice, which lack B and T-cells, and analyzed the IFN-γ and neutrophil response at d7 p.i.. As expected, *Rag2* ^—/—^mice displayed significantly reduced IFN-γ expression (Fig 6D) and increased Ly6G^+^ neutrophil accumulation in the lungs when compared to WT animals (Fig 6E). Together, our findings reveal a mechanism by which IFNs link innate and adaptive immune systems to orchestrate pulmonary inflammation for the resistance to IAV infection.

## Discussion

While it is established that IFN-mediated resistance to viral infection *in vitro* is dependent on the inhibition of viral replication [32], the mechanisms by which IFNs protect against infection *in vivo* are less well understood. Nevertheless, recent studies have suggested that type I IFN signaling is important in regulating myeloid cell migration during viral infections [17,31,33], arguing that IFNs can play a broader role in anti-viral immunity beyond their well-established cell-intrinsic anti-viral activity. In this study, we report that type I IFN signaling is necessary, but not sufficient, to control the full scale of pulmonary innate responses to IAV. To this end, the functional interplay between type I IFN and IFN-γ signaling pathways is required for the regulation of both monocyte- and neutrophil-driven pulmonary inflammation (Fig 6F). The discovery that, in the absence of type I IFN signaling, IFN-γ promotes inflammatory functions of Ly6C^lo^ Mo/Mϕ suggests that the interplay between innate and adaptive IFNs dictates the outcome of tissue inflammation for the resistance to IAV infection.

The impaired Ly6C^hi^ monocyte migration observed in infected *Ifnar1* ^*—/—*^mice bears similarities with *Ccr2* ^*—/—*^mice [10,18,34,35]. Indeed, the CCR2 ligands CCL2, CCL7 and CCL12 are all type I IFN-inducible and influence monocyte recruitment in a model of chronic inflammation [36]. A key difference between *Ccr2* ^*—/—*^and *Ifnar1* ^*—/—*^mice, however, is that CCR2 deficiency is favourable for infection outcome [10,18] whereas *Ifnar1* ^*—/—*^deficiency leads to enhanced mortality compared to WT animals [17]. Therefore, type I IFNs must mediate other protective mechanisms beside the recruitment of Ly6C^hi^CCR2^+^ inflammatory monocytes in IAV infection.

Unexpectedly, we discovered that type I IFN signaling plays a major role in suppressing mononuclear cell activation following IAV infection. Interestingly, this inhibition is particularly effective at suppressing the pro-inflammatory potential of Ly6C^lo^ Mo/Mϕ. Our observation that *Ifnar1* ^—/—^Ly6C^lo^ Mo/Mϕ express higher levels of CD11c, MHC class II and NOS2 than their Ly6C^hi^ (WT or *Ifnar1* ^*—/—*^) counterparts is unexpected. These Ly6C^lo^ cells resemble both phenotypically and functionally the inflammatory monocytes or TNF/iNOS-producing dendritic cells (Tip-DC) described in other inflammatory conditions (reviewed in [12]). Although Ly6C^hi^ and Ly6C^lo^ monocytes are considered to be phenotypically distinct lineages, and their developmental maturation is still under contention [37,38], our data demonstrate that both subsets can be activated by IFN-γ to mediate pro-inflammatory actions. Given these findings, it remains to be established whether CD11b^+^Ly6C^lo^ Mo/Mϕ in lungs of IAV-infected mice represent a separate lineage or have developed from Ly6C^hi^ monocytes as a result of the down-regulation of Ly6C expression upon entry into inflamed lung tissues as described in other models [33,36]. However, the latter scenario is unlikely to be the major mechanism accounting for Ly6C^lo^ Mo/Mϕ accumulation in the absence of type I IFN signaling in our study, because of the significant difference in the numbers of total CD11b^+^ Mo/Mϕ in the lungs of infected WT and *Ifnar1* ^—/—^mice.

Our finding that type I IFNs differentially regulate neutrophil and monocyte trafficking in IAV infection is consistent with a previous report [17]. However, while the previous study analyzed exclusively the cells in the BAL, our investigation examined myeloid populations in both the BALF and lung tissues. Furthermore, in contrast to Seo et al, we did not observe any major changes in progenitor or mature myeloid cells in the BM and blood of IAV-infected *Ifnar1* ^—/—^or mixed BM chimeric mice, suggesting that the IFNs play a major role in coordinating regional immunity rather than central myelopoiesis as proposed by Seo and colleagues [17]. This discrepancy may be explained by the fact that the previous study analyzed myeloid progenitor populations following infection of BM with IAV *in vitro*, an event not typically known to occur during natural infection [39].

Importantly, in addition to defining the function of type I IFNs, we have uncovered two novel functions of IFN-γ in the pulmonary response to IAV infection; the inhibition of neutrophil migration and the induction of monocyte activation. Interestingly, although IFN-γ is known to elicit direct anti-viral activity in infected cells [16] and is produced in high quantities following influenza infection, its function in IAV infection has been elusive. Numerous studies investigating lymphocyte function, viral clearance or survival of mice deficient in IFN-γ or IFN-γR1 collectively reported no appreciable differences compared to WT animals [20,40–42]. Therefore the current study reveals a functional role for IFN-γ in influenza infection and suggests that some IFN-γ functions are masked by type I IFN-dependent regulatory mechanisms. The role of type III IFNs in pulmonary inflammation was not examined in this report, it is possible that the cytokines also play a role in controlling innate cell trafficking and activation during IAV infection. Indeed, a recent study revealed that type III IFNs can regulate neutrophil migration and function in an experimental arthritis model [43]. Interestingly, while type III IFNs are shown to mediate immunity to viral infections, there exists a large degree of redundancy with type I IFNs [44–46]. Future studies investigating the involvement of type III IFNs in this process may provide further insight into the regulatory functions of the IFN system.

Type I IFN production has previously been reported to suppress IFN-γ driven immune responses and resistance to intracellular bacteria [25,26], but it is unknown whether a similar mechanism is activated in viral infection. We demonstrate in this study that the IFN regulatory circuit also plays a pivotal role in the host response to IAV infection, particularly in preventing Ly6C^lo^ Mo/Mϕ from IFN-γ-induced activation by regulating IFN-γR1 expression. The tightly controlled IFN-γ signaling in Ly6C^lo^ Mo/Mϕ may explain why under some circumstance this monocyte population differentiates into alternatively activated macrophages [24], a process known to be susceptible to IFN-γ suppression [47]. Interestingly, the type I IFN cross-regulatory mechanism described here appears to function more actively in some components of the immune response to IAV infection than others, as the inhibitory effect of IFN-γ on neutrophil migration is not suppressed during IAV infection. Nevertheless, this and previous studies collectively suggest that inhibition of IFN-γ function by type I IFN signaling is an important regulatory mechanism operating under some infection and inflammatory settings, where both type I and II IFNs are produced [48]. We propose that in contrast to intracellular bacterial infection, where activation of infected monocytes by IFN-γ is essential for pathogen clearance, inhibition of IFN-γ by type I IFNs during influenza infection serves a host-protective role. Indeed, IFN-γ-inducible nitric oxide (NO) has been shown to play a major role in mediating pulmonary pathology in IAV infection [13,49–51] and, as such, must be tightly controlled to limit immune-mediated tissue damage.

IFNs are potently induced by viral pathogens and mediate host immunity to infections. In this study, we demonstrate the cooperation and cross-regulation between type I and II IFN signaling pathways coordinate a multifaceted pulmonary inflammatory response to IAV infection. Interestingly, it is known that viruses have evolved mechanisms to counteract the host IFN system [52]. Therefore, our findings suggest that in addition to impairing cell-intrinsic anti-viral effector functions as proposed previously [53], blockade of type I IFN production or signaling by viral products may lead to dysregulated inflammation, thereby contributing to impaired disease resistance and possibly increased viral transmission. This hypothesis may explain why some highly virulent strains of IAV are associated with hyper-inflammatory responses [54,55] and suggests that targeted manipulation of IFN signaling pathways could lead to new therapeutic opportunities.

## Materials and Methods

### Mice

C57Bl/6 (CD45.2^+^) and CD45.1^+^ (B6.SJL-Ptprca) mice were obtained from the Animal Resources Centre (ARC, Perth). *Rag2* ^*—/—*^, *Ifnar1* ^*—/—*^, *Ifngr1* ^*—/—*^and *Stat1* ^*—/—*^mice (all on C57Bl/6 background) were bred and maintained at the University of Sydney Bosch Rodent Facility.

### Ethics statement

All mouse work was performed according to ethical guidelines as set out by the University of Sydney Animal Ethics Committee. All experiments within this manuscript were approved under protocol numbers 2013/5847 and 2013/5848. University of Sydney Animal Ethics Committee guidelines adhere to the Australian Code for the Care and Use of Animals for Scientific Purposes (2013) as set out by the National Health and Medical Research Council of Australia.

### Influenza A virus infection

Mice were anaesthetized by intraperitoneal (i.p.) injection with 2% 2-2-tribromoethanol (Avertin) and inoculated intranasally (i.n.) with 20 plaque forming units (PFU) of influenza A virus strain PR8 (A/Puerto Rico/8/1934 H1N1) in a volume of 50 μl. PR8 virus was a kind gift from A/Professor John Stambas (Deakin University, VIC., Australia).

### Influenza A virus plaque assays

IAV was quantified by plaque assays with MDCK cells using standard methods. In brief, 0.9 x 10^6^ MDCK cells were seeded in each well of a 6-well culture plate. Lungs were homogenized in RPMI and clarified by centrifugation for 5 minutes at 2,000 g. Homogenates were serially diluted in RPMI and added to MDCK cell monolayers. After incubation for 45 minutes at 37°C, cells were overlaid with 1% w/v Avicel (FMC Biopolymer) in L15 media (Sigma Aldrich) containing 2 μg/ml TPCK-treated trypsin (Worthington Biochemicals) and incubated at 37°C, 5% CO2 for 3 days. Cells were subsequently washed, methanol fixed and stained with crystal violet before plaques were counted.

### Cell isolation

Euthanized animals were perfused with 10 ml PBS and the lungs removed into 1 ml cold 2% FCS/RPMI. Single cell suspensions were made by dissociating the lungs with a scalpel blade and then incubated in 2% FCS/RPMI supplemented with 2 mg/ml of DNaseI (Sigma Aldrich) and Collagenase IV (Sigma Aldrich) for 30 minutes at 37°C. The digested lungs were then dissociated through a 70 μm cell strainer (Falcon) and red blood cells lysed with ACK lysis buffer (Life Technologies). Cells were counted using trypan blue exclusion.

For bone marrow cells, femurs and tibias were removed and cleaned of flesh before the bone marrow was flushed out with 2% FCS/RPMI. Cells were pelleted at 300 g for 5 minutes and resuspended in ACK lysis buffer (Life Technologies) for 1 minute to lyse red blood cells. Cells were washed and resuspended in 2% FCS/RPMI before being counted by trypan blue exclusion.

For BAL collection, a catheter attached to a 3-way stop cock and 5 ml syringe was inserted into the trachea and the lungs flushed with 5 x 1 ml of cold PBS supplemented with 2 mM EDTA. Cells were pelleted at 300 g for 5 minutes and the cells resuspended in 250 ul of 2% FCS/RPMI and stored at 4°C until used for flow cytometric analysis.

### Generation of bone marrow chimeric mice

BM chimeras were generated by lethally irradiating CD45.1^+^ recipient mice with 10 Gy, followed by intravenous transfer of 2 x 10^6^ BM cells from *Ifnar1* ^*—/—*^mice into the tail vain. For mixed BM chimeras, irradiated CD45.1^+^ recipient WT mice were reconstituted with a total of 2 x 10^6^ BM cells from WT (CD45.1^+^) and *Ifnar1* ^*—/—*^(CD45.2^+^) mice at a ratio of 1:1. Mice received antibiotic-supplemented (Trimethoprim sulpha) drinking water for 3 weeks after irradiation. Mice were used at least 8 weeks after bone marrow reconstitution.

### *In vivo* IFN-γ neutralization in BM chimera mice

For *in vivo* IFN-γ neutralization, mice received 500 μg of anti-IFN-γ monoclonal antibody XMG1.2 intravenously on days 2 and 6 post IAV infection. Anti-IFN-γ clone XMG1.2 hybridoma was expanded in 10% FCS/RPMI supplemented with Pen/Strep (Gibco) and affinity purified using protein G beads (GE Healthcare). Purified antibody was dialyzed into PBS and filter sterilized prior to injection into mice.

### Flow cytometry

Lung (1 x 10^6^), blood (200 ul whole blood) or BM (4 x 10^6^) cells were stained according to standard procedures. Briefly, cells were incubated for 30 minutes with UV Live/Dead stain according to the manufacturer’s instructions (Life Technologies). Cells were stained with the following antibodies in FACS wash (2% FCS/PBS): CD4 (clone GK1.5), CD8 (53–6.7), B220 (RA3-6B2), I-A/I-E (M5-114.15.2), Ly6G (1A8), Ly6C (HK1.4), CD11b (M1/70), CD45.1 (A20), CD45.2 (104), NK1.1 (PK136), Siglec-F (E50-2440), CCR2 (475301), LFA-1 (M17/4), CD119 (2E2), CD11c (N418), CD16/32 (93), CD64 (X54-5/7.1), CD45 (30-F11), Lineage cocktail, CD117 (2B8), Sca1 (D7), CD48 (HM48-1), CD150 (Q38-480). The gating strategy used for identifying cell populations is shown in S6 Fig. Monocyte and macrophage populations were identified as CD4^—^CD8^—^B220^—^NK1.1^—^SiglecF^—^Ly6G^—^CD11b^+^ and then categorized into Ly6C^hi^ and Ly6C^lo^ populations based on Ly6C expression. Neutrophils were identified as CD4^—^CD8^—^B220^—^NK1.1^—^SiglecF^—^CD11b^+^Ly6G^+^.

For intracellular NOS2 staining, single cell suspensions were incubated at 37°C for 3 hours prior to staining with surface marker antibodies. Intracellular staining was carried out using the BD Cytofix/Cytoperm kit according to the manufacturer’s instructions (BD). Intracellular staining was performed using NOS2 antibody (clone CXNFT). All flow cytometry data acquisition was performed on a LSRII using FACSDiva software (BD Biosciences) and all analysis was performed using FlowJo X v0.7 (TreeStar).

### Tissue mRNA preparation and qRT-PCR

Following perfusion, mouse lung tissue was collected and submerged in RNAlater (Ambion) for 24 hours prior to long term storage at -80°C. RNA was prepared from mouse lungs using Trisure (Bioline) according to the manufacturer’s instructions (Bioline). Total RNA (2 μg) was reverse transcribed using the Tetro cDNA synthesis Kit with random primers according to the manufacturer’s instructions (Bioline). Data are expressed as fold increases over uninfected controls and were calculated by the ΔΔCT method using 18S as the reference gene.

For absolute viral nucleoprotein quantification, RNA was extracted from 1 x 10^7^ PFU PR8 using the ISOLATEII RNA kit according to the manufacturer’s instructions (Bioline) and 100 ng reverse-transcribed with the Tetro cDNA synthesis kit using IAV nucleoprotein specific primers [39]. Following amplification, the 216 bp cDNA product was gel purified using a Gel Extraction kit (Sigma Aldrich) and total copy number determined based on size and yield of product. A standard curve was generated to determine absolute viral nucleoprotein mRNA copy number among sample mRNA.

All quantitative reverse-transcriptase PCR (qRT-PCR) was performed using SYBR NoROX master mix (Bioline) on a Roche LightCycler480. Forward and reverse qRT-PCR primers are listed in Table 1.

**Table 1:** List of qRT-PCR primer sequences.

Gene	Forward (5’)	Reverse (3’)
***18S***	GTAACCCGTTGAACCCCATT	CCATCCAATCGGTAGTAGCG
***Cxcl1***	TGTCAGTGCCTGCAGACCAT	CCTCGCGACCATTCTTGAGT
***Il1b***	CAACCAACAAGTGATATTCTCCATG	GATCCACACTCTCCAGCTGCA
***Ifna***	TGCAACCCTCCTAGACTCATT	CCAGCAGGGCGTCTTCCT
***Ifnb***	ATGAGTGGTGGTTGCAGGC	TGACCTTTCAAATGCAGTAGA
***Ifng***	ACAATGAACGCTACACACTGCAT	TGGCAGTAACAGCCAGAAACA
***Ifngr1***	TCAAAAGAGTTCCTTATGTGCCTA	TACGAGGACGGAGAGCTGTT
***IAV NP***	CAGCCTAATCAGACCAAATG	TACCTGCTTCTCAGTTCAAG
***Nos2***	CAGCTGGGCTGTACAAACCTT	CATTGGAAGTGAAGCGTTTCG

### Statistics

All statistical analyses were performed in Prism 6 (GraphPad Software). Significance was determined using Student’s *t*-test when comparing two experimental groups or one-way ANOVA followed by Tukey’s post-test correction for more than two groups. Results with p<0.05 were deemed statistically significant. *<0.05, **<0.01, ***<0.001, ****<0.0001.

## Supporting Information

S1 Fig Sca1 expression is regulated by IFN signaling. Representative flow cytometry plots depicting the proportion of Sca1^+^ cells in the BM of WT and *Stat1* ^—/—^mice at d7 p.i.. Data are representative of 2 independent experiments (n = 4–6 mice).
(TIF)

S2 Fig WT and *Ifnar1* ^—/—^mononuclear cell populations display similar phenotypes early after IAV infection. Representative flow histograms depicting the expression of surface molecules on Ly6C^hi^ and Ly6C^lo^ cell subsets in the lungs of d3 infected WT and *Ifnar1* ^—/—^mice (n = 3 mice / genotype). The flow cytometry data shown are gated on CD11b^+^CD4^—^CD8^—^B220^—^NK1.1^—^Ly6G^—^SiglecF^—^cell populations.
(TIF)

S3 Fig *Ifnar1* ^—/—^Mo/Mϕ show elevated expression of NOS2. **(A)** Representative flow plots showing the percentage of NOS2 and I-A positive CD45.1^+^ (WT) and CD45.2^+^ (*Ifnar1* ^—/—^) Ly6C^hi^ monocytes in the lungs of mixed BM chimera mice at d7 p.i.. The flow cytometry data shown are gated on CD11b^+^CD4^—^CD8^—^B220^—^NK1.1^—^Ly6G^—^SiglecF^—^cell populations. The mixed BM chimeric mice were generated, infected and analyzed as described in Fig 2D. Data are representative of 2 independent experiments (n = 5). **(B)** Paired analysis of mean fluorescence intensity (MFI) of NOS2 in Ly6C^hi^ and Ly6C^lo^ populations of infected mixed BM chimera mice at d7 p.i.. Data were pooled from 2 independent experiments. Statistical analyses were performed using paired Student’s *t*-test.
(TIF)

S4 Fig Percentage of Ly6G^+^ neutrophils in the blood of naive mixed BM chimera mice. The mixed CD45.1^+^ (WT) and CD45.2^+^ (*Ifnar1* ^—/—^) BM chimeric mice were generated, infected and analyzed using flow cytometry as described in Fig 2D. The data show the percentage of CD45.1^+^ (WT) and CD45.2^+^ (*Ifnar1* ^—/—^) Ly6G^+^ neutrophils in the blood of naive mixed BM chimeric mice. Data are representative of 2 independent experiments (n = 10–14 mice / study).
(TIF)

S5 Fig Impaired monocyte migration in IAV infected *Ifnar1* ^-/-^ chimeric mice. Summary data depicting the percentage of Ly6C^hi^ and Ly6C^lo^ mononuclear cells in the lungs of IAV-infected WT, *Ifnar1* ^—/—^and CD45.1^+^ WT / CD45.2^+^ *Ifnar1* ^—/—^chimera mice d7 p.i.. The *Ifnar1* ^—/—^BM reconstituted chimeric mice were generated, infected and analyzed as described in Fig 6. Data are representative of 2 independent experiments.
(TIF)

S6 Fig Gating strategy used to identify lung cell populations. Representative flow cytometry plots of lung single cell suspensions indicating the gating used to identify major lung leukocyte populations. The flow plots shown were acquired from a day 7 p.i. WT mouse.
(TIF)
