# Supplementary figures and images for: Functional Interplay between Type I and II Interferons Is Essential to Limit Influenza A Virus-Induced Tissue Inflammation
Source: PLoS Pathog. 2016 Jan 5;12(1):e1005378. doi: 10.1371/journal.ppat.1005378 (PMC4701664; doi:10.1371/journal.ppat.1005378)

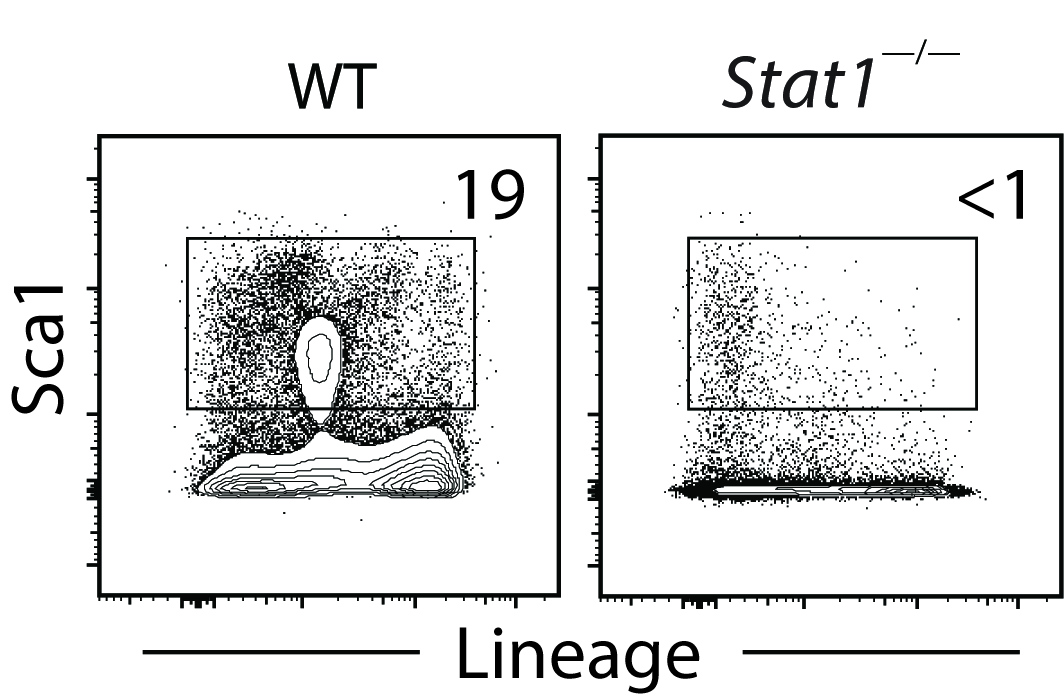

Supplement: S1 Fig — Representative flow cytometry plots depicting the proportion of Sca1+ cells in the BM of WT and Stat1 —/—mice at d7 p.i.. Data are representative of 2 independent experiments (n = 4–6 mice). (TIF) [file ppat.1005378.s001.tif]

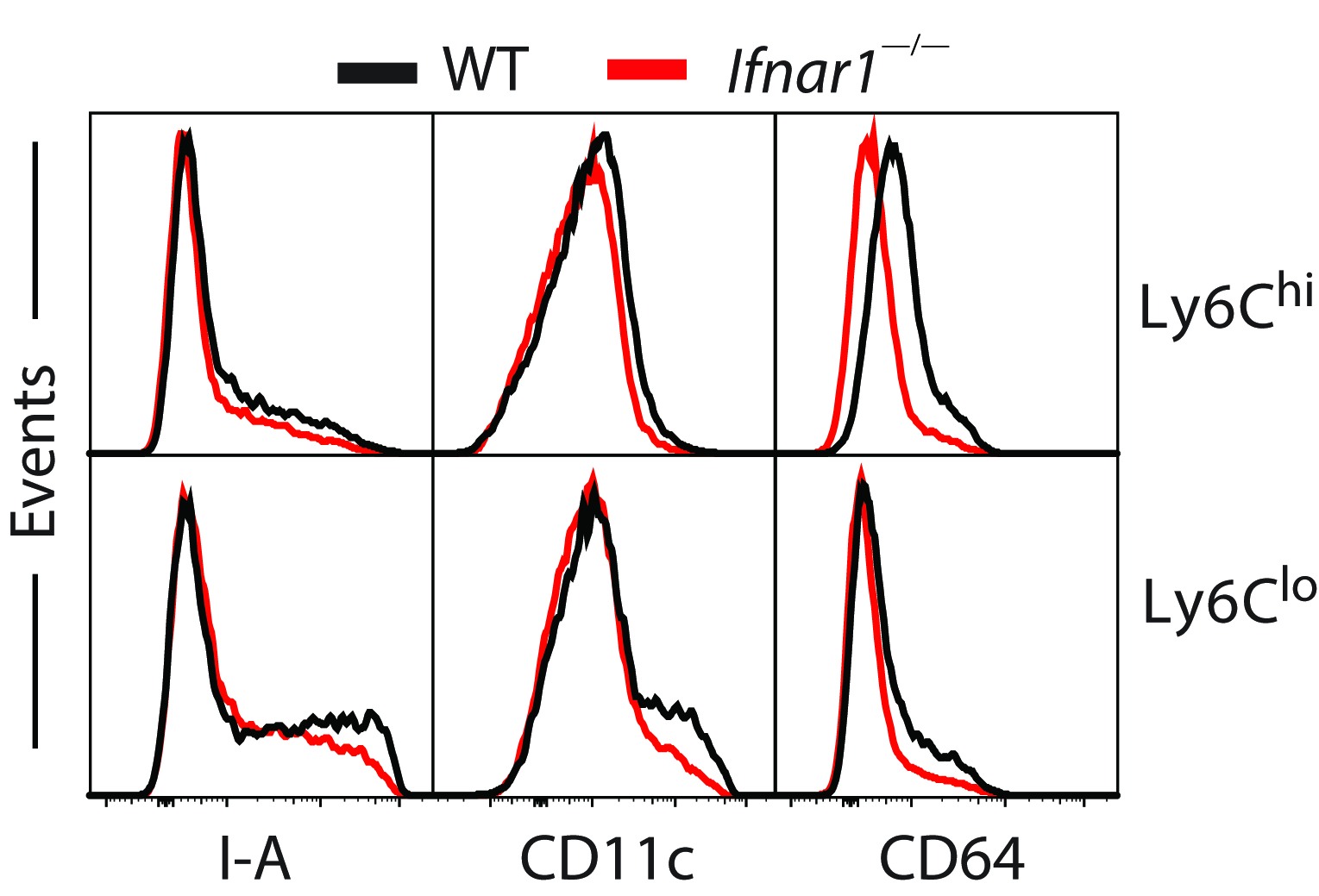

Supplement: S2 Fig — Representative flow histograms depicting the expression of surface molecules on Ly6Chi and Ly6Clo cell subsets in the lungs of d3 infected WT and Ifnar1 —/—mice (n = 3 mice / genotype). The flow cytometry data shown are gated on CD11b+CD4—CD8—B220—NK1.1—Ly6G—SiglecF—cell populations. (TIF) [file ppat.1005378.s002.tif]

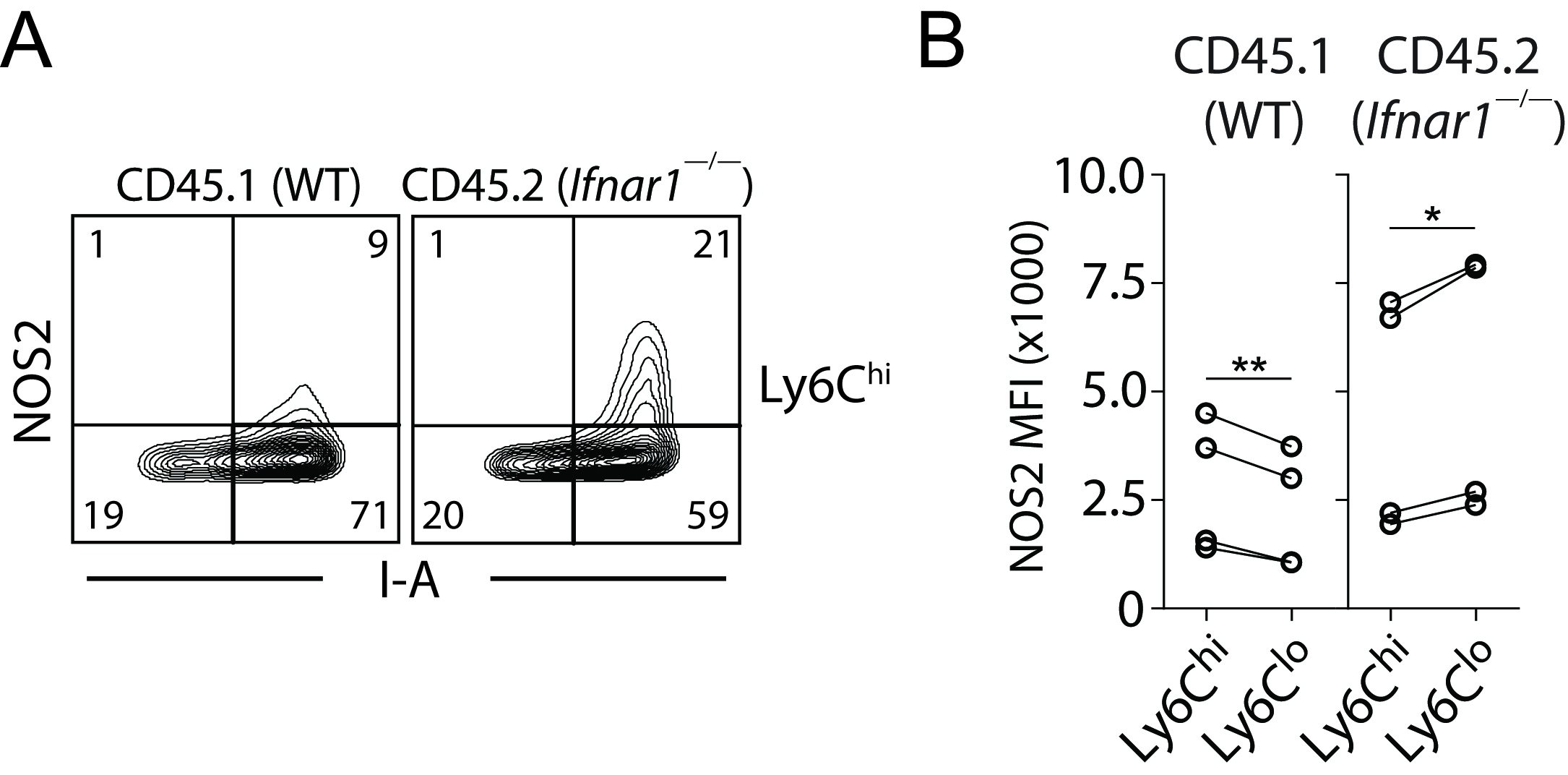

Supplement: S3 Fig — (A) Representative flow plots showing the percentage of NOS2 and I-A positive CD45.1+ (WT) and CD45.2+ (Ifnar1 —/—) Ly6Chi monocytes in the lungs of mixed BM chimera mice at d7 p.i.. The flow cytometry data shown are gated on CD11b+CD4—CD8—B220—NK1.1—Ly6G—SiglecF—cell populations. The mixed BM chimeric mice were generated, infected and analyzed as described in Fig 2D. Data are representative of 2 independent experiments (n = 5). (B) Paired analysis of mean fluorescence intensity (MFI) of NOS2 in Ly6Chi and Ly6Clo populations of infected mixed BM chimera mice at d7 p.i.. Data were pooled from 2 independent experiments. Statistical analyses were performed using paired Student’s t-test. (TIF) [file ppat.1005378.s003.tif]

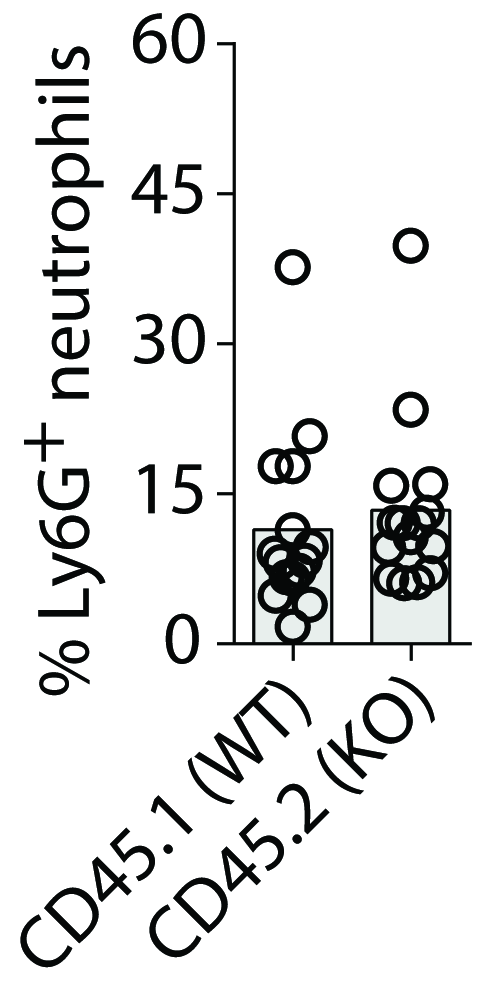

Supplement: S4 Fig — The mixed CD45.1+ (WT) and CD45.2+ (Ifnar1 —/—) BM chimeric mice were generated, infected and analyzed using flow cytometry as described in Fig 2D. The data show the percentage of CD45.1+ (WT) and CD45.2+ (Ifnar1 —/—) Ly6G+ neutrophils in the blood of naive mixed BM chimeric mice. Data are representative of 2 independent experiments (n = 10–14 mice / study). (TIF) [file ppat.1005378.s004.tif]

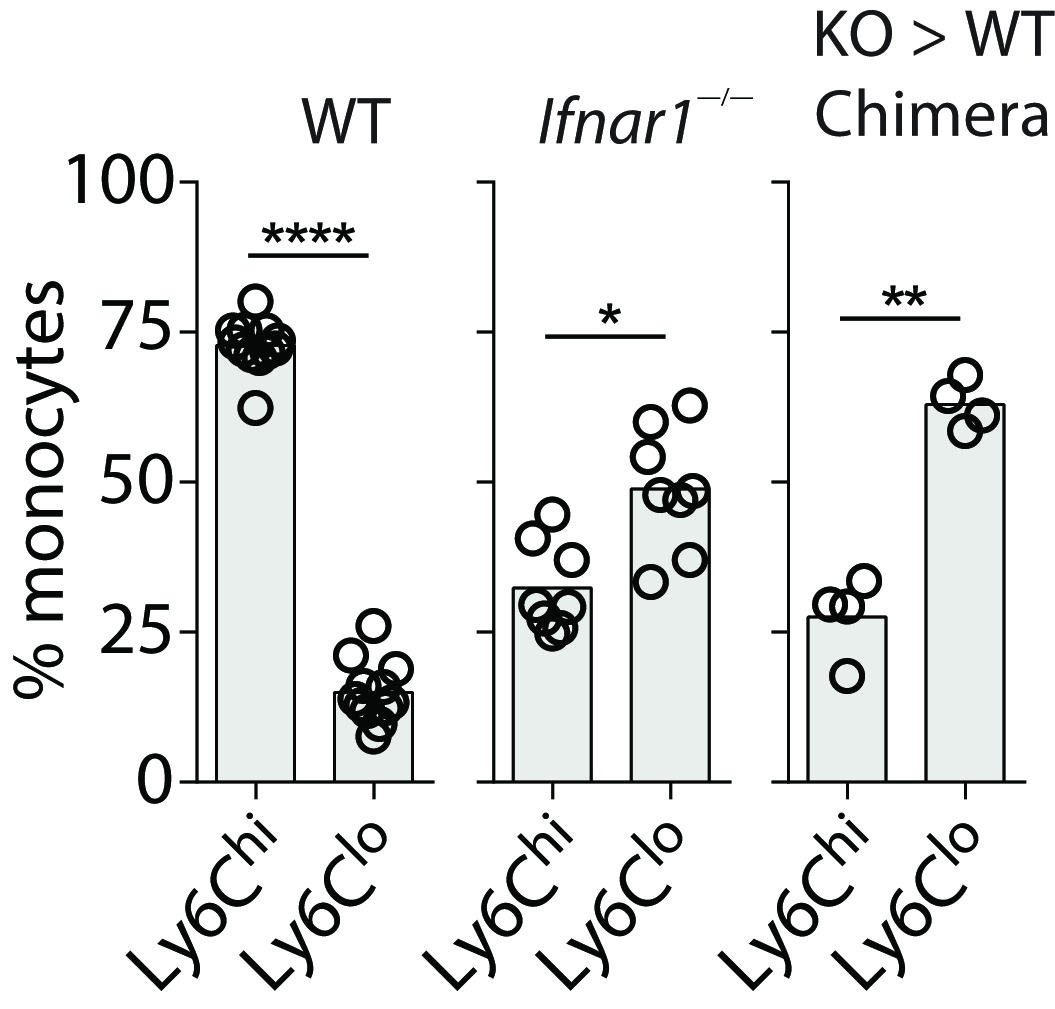

Supplement: S5 Fig — Summary data depicting the percentage of Ly6Chi and Ly6Clo mononuclear cells in the lungs of IAV-infected WT, Ifnar1 —/—and CD45.1+ WT / CD45.2+ Ifnar1 —/—chimera mice d7 p.i.. The Ifnar1 —/—BM reconstituted chimeric mice were generated, infected and analyzed as described in Fig 6. Data are representative of 2 independent experiments. (TIF) [file ppat.1005378.s005.tif]

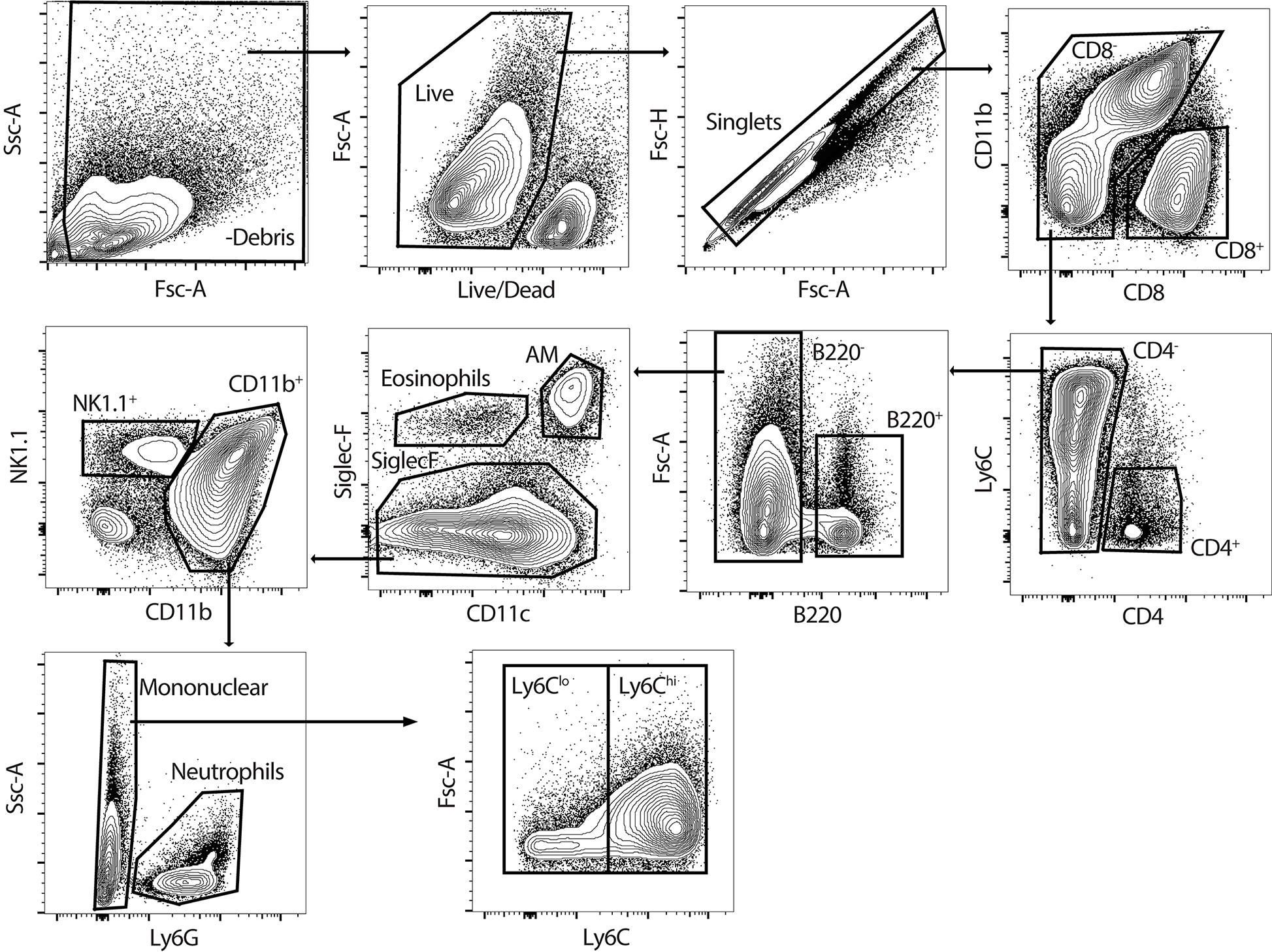

Supplement: S6 Fig — Representative flow cytometry plots of lung single cell suspensions indicating the gating used to identify major lung leukocyte populations. The flow plots shown were acquired from a day 7 p.i. WT mouse. (TIF) [file ppat.1005378.s006.tif]
